# Optimal Use of Plant Stanol Ester in the Management of Hypercholesterolemia

**DOI:** 10.1155/2015/706970

**Published:** 2015-10-12

**Authors:** Susanna Rosin, Ilkka Ojansivu, Aino Kopu, Malin Keto-Tokoi, Helena Gylling

**Affiliations:** ^1^Raisio Group, Benecol Unit, P.O. Box 101, FI-21201 Raisio, Finland; ^2^University of Helsinki and Helsinki University Hospital, Internal Medicine, P.O. Box 700, 00029 Helsinki, Finland

## Abstract

Plant stanol ester is a natural compound which is used as a cholesterol-lowering ingredient in functional foods and food supplements. The safety and efficacy of plant stanol ester have been confirmed in more than 70 published clinical studies and the ingredient is a well-established and widely recommended dietary measure to reduce serum cholesterol. Daily intake of 2 g plant stanols as plant stanol ester lowers LDL-cholesterol by 10%, on average. In Europe, foods with added plant stanol ester have been on the market for 20 years, and today such products are also available in many Asian and American countries. Despite the well-documented efficacy, the full potential in cholesterol reduction may not be reached if plant stanol ester is not used according to recommendations. This review therefore concentrates on the optimal use of plant stanol ester as part of dietary management of hypercholesterolemia. For optimal cholesterol lowering aiming at a lower risk of cardiovascular disease, plant stanol ester should be used daily, in sufficient amounts, with a meal and in combination with other recommended dietary changes.

## 1. Introduction

Atherosclerotic vascular diseases are the most common cause of mortality worldwide. High cholesterol induces a third of all cardiovascular disease, causing about 17 million deaths per year [1]. Lowering LDL-cholesterol is a central target in the prevention of cardiovascular diseases, especially coronary heart disease. It is estimated that for every 1% reduction in LDL-cholesterol concentration, there is a corresponding 1 to 2% decrease in the risk of coronary heart disease [2, 3]. Recent research findings further emphasize the importance of LDL-cholesterol management in the prevention of cardiovascular diseases [4, 5].

Atherosclerosis develops during a long period of time so the earlier the lipid management is initiated the more likely the atherosclerotic vascular diseases can be prevented [6]. Since atherosclerosis is largely a result of an unhealthy lifestyle, lifestyle intervention should be favored in its prevention and management. Central international prevention and treatment guidelines therefore stress the importance of diet and lifestyle as the cornerstones of hypercholesterolemia management [7–9]. A big advantage of this approach is that lipid management can be safely implemented from early age.

Permanent changes in lifestyle may be hard to achieve, however, and people with elevated cholesterol levels may not always be motivated enough to make the recommended changes [10]. One solution is to use cholesterol-lowering functional foods and food supplements which offer an easy and convenient way of lowering cholesterol through diet.

Plant stanol ester is one of the most established cholesterol-lowering functional food ingredients in the world. Its efficacy has been verified in more than 70 published clinical studies, and its use as part of a cholesterol-lowering diet is recommended by a number of international nongovernmental and scientific organisations [7–9]. However, permanent reductions in serum cholesterol can be achieved only through sufficient daily use of plant stanol ester as part of daily meals. This review thus concentrates on highlighting the correct use of plant stanol ester as part of a healthy lifestyle.

## 2. What Is Plant Stanol Ester?

Plant stanols and plant sterols are natural compounds which are present in our daily diet. Their main dietary sources are vegetables, fruit, vegetable oils, cereals, and nuts [11]. A western diet typically contains 20 to 30 mg/d plant stanols and around 300 mg/d plant sterols [12, 13]. However, this intake from naturally occurring sources is too low to bring about a significant cholesterol-lowering effect, which is why plant stanols and plant sterols are added to foods and food supplements.

Plant stanols are added to commercially available cholesterol-lowering products as plant stanol ester. This fat-soluble compound is formed of plant stanols and vegetable oil based fatty acids through esterification. Adding plant stanols as plant stanol ester ensures the cholesterol-lowering efficacy of the ingredient without altering the taste or the texture of the food product.

### 2.1. Mode of Action

Plant stanol ester works by partly inhibiting the absorption of cholesterol in the small intestine. Plant stanol ester is first hydrolysed to plant stanols and fatty acids. After this, plant stanols interfere with the solubilisation of cholesterol, that is, the incorporation of cholesterol into mixed micelles [14]. This is possible because of the slight structural dissimilarities of plant stanols and cholesterol. Other mechanisms, such as activation of certain transport proteins in the enterocytes, may also be involved in the process. Plant stanols themselves are virtually not absorbed but are rapidly excreted from the body in feces [15].

Consumption of plant stanol ester inhibits the absorption of both dietary cholesterol, that is, the cholesterol coming to the digestive tract in food, and biliary cholesterol, that is, the cholesterol coming to the digestive tract in bile. With 2 g/d plant stanols, cholesterol absorption efficiency is reduced by about 50% [16, 17]. Optimal cholesterol absorption inhibition requires that plant stanol ester is consumed with a meal because bile is excreted into the digestive tract following a meal. The reduced cholesterol absorption achieved with plant stanol ester leads to significantly reduced levels of serum total and LDL-cholesterol [16, 18–21].

### 2.2. Efficacy in LDL-Cholesterol Lowering

Plant stanol ester is one of the most established functional food ingredients in the world. Research evidence consistently shows that a daily intake of 2 g of plant stanols (as plant stanol ester) reduces total and LDL-cholesterol concentration by 10%, on average [22, 23], with no effect on HDL-cholesterol concentration [24–26].

**Figure 1:**
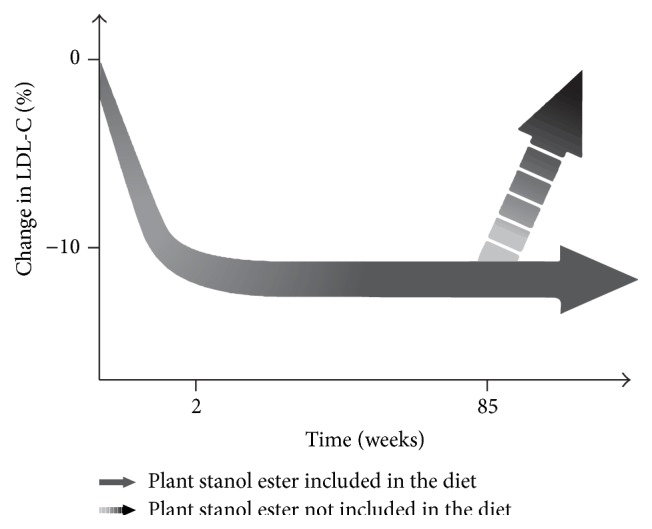
The full LDL-cholesterol-lowering effect of plant stanol ester can be detected after the daily consumption of 2 g/d plant stanols for 2 weeks. The effect is sustained as long as plant stanol ester is included in the daily diet; the longest intervention period in a clinical study has been 85 weeks. If the consumption is stopped, the plant stanol ester specific cholesterol-lowering effect will be lost within 1-2 weeks (modified from [18, 22, 24, 27, 32, 33, 35–37]).

The average 10% reduction in LDL-cholesterol concentration can be detected after only 1 to 2 weeks' daily consumption of plant stanol ester (2 g/d plant stanols) [27–31]. More important, however, is that the reduction in blood LDL-cholesterol is persistent as long as plant stanol ester is included in the daily diet (Figure 1). The study by Miettinen et al. (1995) [24] was the first long-term study showing that 1.8–2.6 g/d plant stanols as part of a daily diet effectively reduced LDL-cholesterol concentration by 12–14% in a mildly hypercholesterolemic population. The intervention period in this study lasted for 12 months. The long-term effect of plant stanol ester has been confirmed in other studies, in which the intervention period has lasted from 12 months to 85 weeks [32, 33]. It is noteworthy that when the daily consumption of plant stanol ester is stopped, serum LDL-cholesterol concentration rapidly increases towards initial levels [24].

### 2.3. Safety

Studies on plant stanol ester have been conducted in several population groups, including normocholesterolemic or mildly or moderately hypercholesterolemic adults and children, individuals with familial hypercholesterolemia, individuals with type 1 or type 2 diabetes, and patients with coronary heart disease. These studies have included subjects from Europe, Americas, and Asia [38]. No adverse effects have been reported in connection with the use of plant stanol ester in the above-mentioned clinical studies or in regular consumption of plant stanol ester containing products.

Plant stanols are stable molecules that are not altered in food manufacturing or preparation processes. Being microbiologically inert they are not effected by fermentation [39] nor are they oxidized when heated [40]. Likewise they are not metabolized in the body but excreted intact [15].

The United States Food and Drug Administration (U.S. FDA) has acknowledged the GRAS status (Generally Recognised as Safe status) of plant stanol ester [41], and the safety of plant stanol ester has been evaluated by local food safety authorities before entering the respective markets. Furthermore, the Joint FAO/WHO Expert Committee on Food Additives (JECFA) has considered plant stanol ester as safe [39].

## 3. Optimal Use of Plant Stanol Ester

The key to successful serum cholesterol reduction with plant stanol ester is the correct use of this ingredient. Since hypercholesterolemic patients typically discuss their diet with healthcare professionals, the latter need to be informed on the optimal use of plant stanol ester. Key considerations that need to be taken into account when plant stanol ester is used to manage hypercholesterolemia are discussed below.

### 3.1. Sufficient and Daily Consumption

Most of the clinical studies assessing the cholesterol-lowering effect of plant stanol ester have been conducted with a 2 g/d intake [23] and research evidence consistently shows that a daily intake of 2 g plant stanols lowers serum LDL-cholesterol concentration by 10%, on average [22]. International guidelines also encourage clinicians to consider this daily intake of plant stanols (2 g/d) as part of an overall healthy diet [7–9]. This recommended intake is also communicated to consumers in food labeling.

Some recent research indicates that further cholesterol reduction can be obtained with higher doses than those currently recommended, and also the safety of plant stanol ester has been confirmed with daily intakes of plant stanols up to 9 g [23, 42, 43].

Despite clear consumer communication, the average intake of plant stanols from enriched products is typically well below the recommended 2 g/d. The same concerns plant sterols. For example, a Finnish study showed that more than half of the users of foods with added plant stanol or plant sterol ester did not reach the recommended daily intake [44]. Another example is a survey on consumer purchase behaviour which included 91,000 households in the Netherlands, Belgium, United Kingdom, France, and Germany [45]. According to this survey, the average intake of plant stanols or sterols was only 0.35–0.86 g/day.

A too low daily intake of plant stanols or plant sterols is problematic since the expected cholesterol-lowering effect may not be reached. Based on a series of meta-analyses which were published in a rebuttal by Musa-Veloso and Poon [46] it can be calculated that daily intakes of 1.0, 1.5, and 2.0 grams of plant stanols will bring about average reductions of 5.3%, 7.4%, and 9.1%, respectively, in serum LDL-cholesterol concentration. Thus, the effect of daily intake of 1.0 grams or less may easily be hidden behind the natural variation in serum cholesterol concentration. Furthermore, the European Food Safety Authority, EFSA, has concluded that 1.5–2.4 g/d plant stanols or plant sterols are needed in order to reach a significant 7–10% reduction in serum LDL-cholesterol [47].

The low plant stanol intake may be due to irregular use of foods with added plant stanol ester or too small daily portions of such foods. Therefore, it is important that healthcare professionals emphasize the sufficient daily consumption of plant stanol ester when they discuss diet with their patients.

### 3.2. Best Efficacy When Consumed with a Meal

For optimum efficacy, it is important to consume foods and food supplements with added plant stanol ester with daily meals [25, 48, 49]. This is due to the mechanism of action of plant stanol ester, where plant stanols partly replace cholesterol in mixed micelles which are formed in the digestive tract. Micelle formation requires sufficient amounts of fat and other macronutrients, and without a meal the daily plant stanol ester dose may bring only around 50% of optimal efficacy [50].

Also the esterification of plant stanols to plant stanol ester is* per se* an efficient way of ensuring that plant stanols are effectively incorporated into mixed micelles for effective reduction of cholesterol absorption [51]. Consumption with a meal, however, ensures that bile is excreted to the digestive tract and that the reabsorption of biliary cholesterol can be inhibited with plant stanol ester.

Most studies have assessed the effects of taking 2 grams of plant stanols in two or more portions per day. However, taking the daily dose of plant stanols in one dose with a main meal has been shown to be as effective in lowering serum LDL-cholesterol concentrations as splitting the dose over three meals [48].

### 3.3. Additive Effect to Other Cholesterol-Lowering Dietary Measures

Plant stanol ester effectively reduces serum total and LDL-cholesterol concentration as part of any diet. It works in a typical western diet with a relatively high content of saturated fat and cholesterol [24] as well as diets low in saturated fat and cholesterol [26, 37, 52, 53]. Thus, the average 10% reduction in LDL-cholesterol is independent of the background diet.

Since the effect of plant stanol ester is additive to other cholesterol-lowering dietary changes [37, 52], it should optimally be consumed as part of an overall heart-healthy diet. A recommended diet including plant stanol ester can reduce LDL-cholesterol by up to 20–30% (Table 1) [7, 34, 37].

### 3.4. Additive Effect to Statins

International guidelines recommend the use of plant stanol ester also in combination with cholesterol-lowering statin medication [7–9]. These two cholesterol-lowering means have different mechanisms of action, and consequently the cholesterol-lowering effect of plant stanol ester adds to the effect of statins. This additive effect has been shown in several randomized, controlled clinical trials where patients on stable statin medication have included foods with added plant stanol ester in their diet, resulting in an incremental LDL-cholesterol reduction of 10%, on average [17, 33, 35, 54–58]. This 10% reduction achieved with plant stanol ester is larger than that of doubling the statin dose [59].

## 4. For Which Patients Should Plant Stanol Ester Be Considered?

Recently, a Consensus Panel of the European Atherosclerosis Society published a Consensus Statement on the role of plant stanols and plant sterols in the management of dyslipidemia and prevention of cardiovascular disease [14]. This Consensus Statement as well as many other international guidelines [7–9, 60–64] encourages clinicians to consider the use of functional foods with added plant stanols (2 g/d) as an aid to other beneficial lifestyle changes, also in conjunction with cholesterol-lowering medication. Key patient groups for which the daily use of plant stanol ester can be considered are listed as an adjunct to a general healthy diet and lifestyle [7–9, 14, 61–64] as follows:

1. individuals who have elevated serum cholesterol levels and are at low or intermediate global cardiovascular risk but who do not need cholesterol-lowering medication,
2. high and very high risk patients, such as patients with diabetes, who fail to achieve LDL-cholesterol targets on statins alone or are statin intolerant,
3. adults and children (from the age of 6 years) with familial hypercholesterolemia.

## 5. Conclusions

Atherosclerotic cardiovascular events are still the number one killer in the world. However, if LDL-cholesterol level was kept low throughout life or if high LDL-cholesterol was effectively lowered, the development of atherosclerosis could be prevented or the progress of atherosclerotic plaque formation reversed. Diet plays a central role in the life-long management of blood cholesterol levels, and because recommended dietary changes are an inexpensive, safe, and effective way to reduce cholesterol, all dietary measures, including the use of functional foods with added plant stanol ester, should be fully utilized. However, the challenge is that patients may find it hard to motivate themselves to make permanent changes to their diet and lifestyle. Plant stanol ester is a well-documented and safe dietary way to reduce cholesterol. When used daily in sufficient amounts and as part of the daily meals, it can reduce serum LDL-cholesterol by on average 10%. In combination with other recommended dietary changes, the total reduction in serum LDL-cholesterol can be as much as 20–30%. Healthcare professionals should actively communicate the efficacy of a recommended cholesterol-lowering diet to their hypercholesterolemic patients to increase the patients' motivation for dietary changes.

## Figures and Tables

**Table 1:** Key dietary interventions to reduce serum total and LDL-cholesterol levels (modified from [7, 34]).

Dietary intervention	Average reduction in LDL-cholesterol
Replacement of saturated and trans fat with unsaturated fats	5–10%
Increase in dietary fibre intake	5%
Utilization of functional foods with added plant stanols	10–15%
Cumulative estimate	**20**–**30%**
